# A model based on dynamic hematologic parameters to predict short term clinical events in pediatric acute lymphoblastic leukemia

**DOI:** 10.55730/1300-0144.6183

**Published:** 2026-03-04

**Authors:** Neryal TAHTA, Salih GÖZMEN, Sultan OKUR ACAR

**Affiliations:** 1 Department of Pediatric Hematology Oncology, İzmir City Hospital, İzmir, Turkiye; 2 Department of Pediatric Hematology Oncology, Faculty of Medicine, Dokuz Eylül University, İzmir, Turkiye

**Keywords:** Pediatric acute lymphoblastic leukemia, hematologic variability, short-term clinical events, prediction model, neutrophil dynamics, symptom burden, mixed-effects logistic regression, risk stratification

## Abstract

**Background/aim:**

Acute lymphoblastic leukemia (ALL) treatment is frequently complicated by infections, emergency visits, and therapy interruptions, yet early prediction of short-term clinical deterioration remains challenging. Traditional prognostic markers rely on static laboratory values, whereas dynamic hematologic fluctuations may provide earlier warning signals. This study presents and internally validates a clinically applicable prediction model based on dynamic hematologic parameters and clinician-documented symptoms for predicting short-term (7-day) clinical events in children with ALL.

**Materials and methods:**

Included in this retrospective study were 44 pediatric ALL patients treated with Berlin-Frankfurt-Münster-based protocols between January 2023 and June 2025. Weekly observation units were created by aggregating complete blood count values and clinician-documented symptoms. Dynamic hematologic indices included mean absolute neutrophil count (ANC), coefficient of variation (ANC-CV), and time in target range (ANC-TTR). The composite outcome was defined as any of the following occurring within 7 days: unplanned emergency visit, ≥48-h chemotherapy interruption, or infection requiring systemic antibiotics. Mixed-effects logistic regression was used to account for within-patient clustering. Model performance was assessed using discrimination, calibration, decision curve analysis, and bootstrap internal validation.

**Results:**

A total of 1136 weekly observations were analyzed. Composite clinical events occurred in 32.3% of weeks. Event weeks demonstrated lower ANC, higher ANC-CV, reduced ANC-TTR, lower hemoglobin levels, and higher symptom burden (all p <0.01). In the hematology-only model, ANC, ANC-CV, ANC-TTR, hemoglobin levels, and platelet counts were independent predictors (AUROC = 0.77). Adding the symptom score improved discrimination (AUROC = 0.83) and calibration. Decision curve analysis demonstrated greater net clinical benefit for the combined model across threshold probabilities of 10–40%.

**Conclusion:**

Dynamic hematologic trajectories and clinician-documented symptoms enable accurate early prediction of short-term clinical events in pediatric ALL. This low-cost, accessible prediction model may support individualized risk stratification and proactive supportive care.

## 1. Introduction

Acute lymphoblastic leukemia (ALL) is the most common childhood malignancy, accounting for one-third of all pediatric cancers [1]. Collaborative treatment protocols, risk-adapted therapy, and improved supportive care have increased 5-year survival rates to over 85% in many countries [2–4]. Nevertheless, treatment remains prolonged and intensive, and therapy-related toxicities; particularly infectious complications, unplanned healthcare utilization, and chemotherapy interruptions, continue to impose substantial morbidity. Outcomes in countries using Berlin-Frankfurt-Münster (BFM)-based regimens are increasingly comparable to international standards [5,6], yet predicting early toxicity and treatment intolerance remains a persistent clinical challenge.

Traditional prognostic frameworks rely on static diagnostic factors such as age, leukocyte count, cytogenetics, and minimal residual disease (MRD) response [7,8]. Although these indicators are strongly predictive of long-term survival and relapse risk, they offer limited insight into short-term clinical instability occurring during therapy. Emerging evidence suggests that dynamic hematologic parameters, not only the absolute degree of neutropenia but also week-to-week fluctuations in absolute neutrophil count (ANC), hemoglobin, and platelet counts, may provide earlier signals of infectious susceptibility, marrow suppression, or intolerance to chemotherapy [9–13]. However, previous studies have primarily examined single neutrophil thresholds or dose-intensity relationships, leaving the predictive value of temporal variability largely unexplored.

Myelosuppression during ALL treatment is shaped by multiple factors, including host pharmacogenetics, thiopurine metabolism, treatment phase, and adherence to oral maintenance therapy [9,12]. Variability in mercaptopurine and methotrexate metabolism has been associated with unpredictable ANC dynamics, reinforcing the rationale for monitoring hematologic trajectories across treatment cycles [9,11,12]. Several clinical tools have been proposed for the prediction of infectious complications or bacteremia in children with cancer, particularly in cases of febrile neutropenia; however, these models generally rely on static laboratory thresholds and acute clinical presentations rather than longitudinal hematologic variability. Moreover, most available prediction approaches focus on single episodes of febrile neutropenia rather than continuous monitoring of short-term clinical instability throughout ALL therapy. Consequently, the clinical utility of dynamic hematologic variability as an early warning signal for short-term deterioration remains insufficiently explored.

In parallel, standardized symptom-based tools such as the Pediatric Patient-Reported Outcomes version of the Common Terminology Criteria for Adverse Events (PRO-CTCAE) aim to quantify treatment burden more accurately [14–17]. However, these instruments are predominantly used in prospective trials, whereas routine clinical documentation remains heterogeneous, while prediction models that integrate both dynamic hematologic data and symptom severity remain scarce.

There is thus a need for practical and data-driven tools capable of identifying short-term clinical risk, such as impending infection, emergency visits, or therapy interruptions, particularly in high-volume settings with limited supportive care resources. The objectives of the present study are threefold: (1) to characterize weekly hematologic dynamics in children with ALL; (2) to determine the association between dynamic hematologic variability and short-term clinical events; and (3) to develop and internally validate a clinically applicable prediction model that takes into account both hematologic variability and clinician-documented symptoms.

By leveraging high frequency laboratory data and clinical information, this study supports a more individualized and proactive approach to toxicity management in pediatric ALL [18,19].

## 2. Materials and methods

Included in this retrospective study were pediatric patients diagnosed with ALL and treated at a tertiary pediatric hematology-oncology center between January 1, 2023 and June 30, 2025. All procedures were conducted in accordance with the principles of the Declaration of Helsinki, and ethical approval for the study protocol was granted by the institutional ethics committee (approval number: 2025/553; date: 05.11.2025).

A total of 44 pediatric patients aged 1–18 years who were treated according to BFM-based national protocols were included in the study. The eligibility criteria were: (i) a confirmed diagnosis of ALL; (ii) availability of at least eight consecutive weeks of complete blood count (CBC) measurements during therapy; and (iii) accessible clinical documentation of weekly events (emergency visits, dose interruptions, infections).

Exclusion criteria were: (i) early relapse requiring planned allogeneic hematopoietic stem cell transplantation; (ii) missing hematologic data; and (iii) continuation of therapy at another institution.

Patient data were accessed from electronic medical records and the institution’s laboratory information system. Weekly observation units were constructed for each patient, and the mean ANC, hemoglobin (Hb), platelet (PLT), white blood cell (WBC), and lymphocyte values were recorded for each week.

Dynamic hematologic indices were calculated as follows: ANC-CV (coefficient of variation) = SD/mean × 100; ANC-TTR (time in target range) = proportion of ANC values between 500–1500/μL within the week. The target ANC range was selected based on clinical practice and previous literature in which values <500/μL are associated with increased infection risk, whereas persistently higher counts may reflect reduced chemotherapy intensity. This range is also commonly used to guide dose adjustments and supportive care during ALL therapy.

The primary endpoint was a composite event occurring within 7 days of a weekly observation, defined as the presence of ≥1 of the following: unplanned emergency visit, ≥48-h chemotherapy interruption or dose reduction, infection requiring systemic antibiotics, or hospitalization.

Clinician documented symptoms (mucositis, nausea/vomiting, fatigue, pain) were extracted from weekly examination notes and recoded using a simplified schema modeled based on Pediatric PRO-CTCAE categories: 0 = absent, 1 = mild, 2 = moderate/severe. The weekly symptom score was the maximum severity documented in the week in question. Symptom extraction was performed by a single investigator using a predefined structured framework to improve consistency.

All analyses were performed using R version 4.3.2. Continuous variables were expressed as mean ± SD or median interquartile range (IQR) and categorical variables as numbers and percentages. Weekly observations of individuals were correlated; therefore, mixed-effects logistic regression with patient ID as a random intercept was employed to evaluate predictors of 7-day clinical events [7].

Dynamic hematologic indices (ANC mean, ANC-CV, ANC-TTR), Hb, PLT, and symptom score were selected based on clinical relevance and previous literature. A hematology-only model (Model 1) and a combined hematology + symptom model (Model 2) were developed. To reduce overfitting, variable shrinkage and selection were guided by LASSO regression.

The precision of variability indices depends on the number of CBC measurements per weekly unit. In our cohort, the median number of CBC measurements per week was 2 (IQR: 1–3). Weeks with fewer than two measurements were infrequent (<10% of observations), and for such weeks, the mean ANC was retained, whereas ANC-CV was not calculated. For ANC-TTR, single measurements were classified as either within or outside the target range. Sensitivity analyses excluding these weeks yielded similar results.

Missing data were minimal due to the routine nature of CBC monitoring in pediatric ALL. The overall proportion of missing values across hematologic variables was <5%. A complete-case analysis approach was performed, as the proportion of missing data was low and assumed to be random.

Multicollinearity among predictors was assessed using correlation matrices and variance inflation factors (VIFs). All VIF values were below 3, indicating no significant multicollinearity.

Model performance was evaluated based on area under the receiver operating characteristic (ROC) curve (AUROC), calibration plots, Hosmer–Lemeshow goodness-of-fit test, Internal validation via bootstrap resampling (1000 iterations), and decision curve analysis (DCA) to assess the net clinical benefit across threshold probabilities [19]. Calibration slope and intercept were also estimated from bootstrap validation. Variable selection was guided by LASSO-penalized logistic regression applied to the fixed-effects structure, followed by construction of the final mixed-effects logistic regression model to account for within-patient clustering. Statistical significance was defined as p < 0.05.

## 3. Results

**Figure 1:**
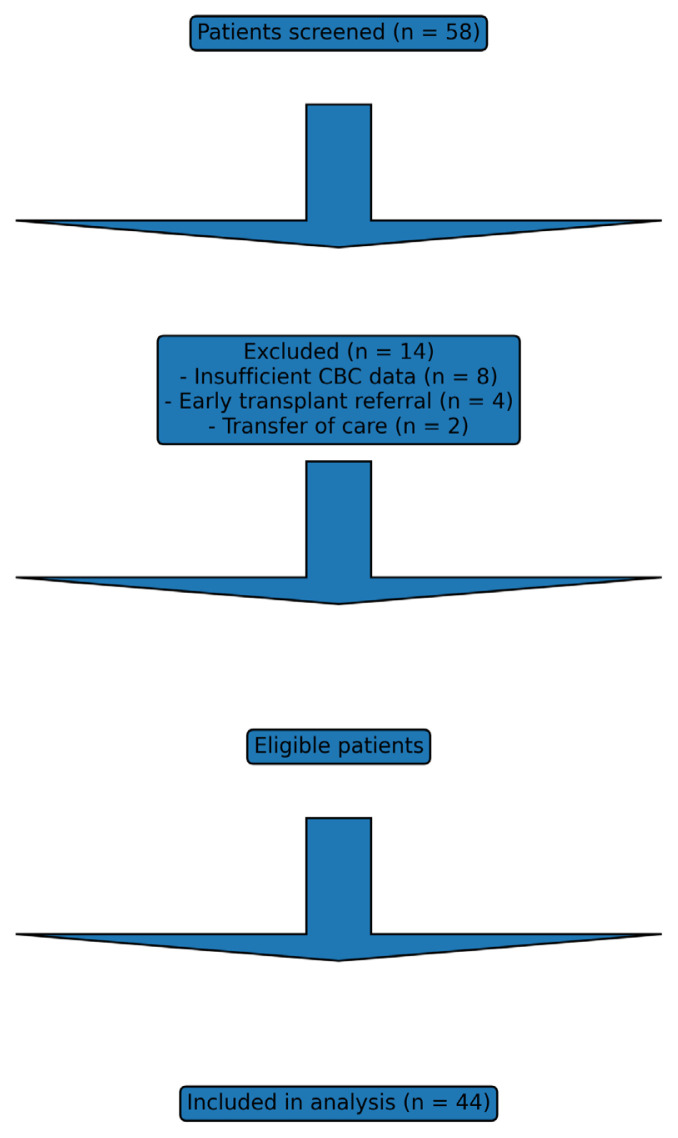
Study flow diagram: patient screening, exclusion reasons, and final cohort included in the prediction model development and validation.

Included in the study were 44 pediatric patients with ALL who met the inclusion criteria. A patient flow diagram detailing the screening, exclusion, and inclusion process is presented in Figure 1. A total of 58 patients were initially screened, of whom eight were excluded due to insufficient CBC data, four due to early referral for transplantation, and two who transferred to another institution. Baseline demographic and clinical characteristics are presented in Table 1. The median age at diagnosis was 6.1 years (IQR 3.3–10.4), and 52.3% were male. B-cell ALL accounted for 84.1% of cases, while 15.9% were T-ALL. Risk stratification based on BFM protocols yielded 45.5% standard-risk, 38.6% intermediate-risk, and 15.9% high-risk disease.

The median follow up duration was 14.1 months (IQR 10.0–19.6). The 2-year observed relapse during follow-up was 9.1% (n = 4), and mortality was 4.5% (n = 2).

A total of 1136 weekly observation units were generated (median 25 per patient). Composite clinical events occurred in 32.3% of weeks (367/1136), and included unplanned emergency room visits (19.7%), chemotherapy interruptions ≥48 h (14.8%), and infections requiring antibiotics or hospitalization (17.4%). Event overlaps were common, with ≥2 events occurring in 42.8% of weeks. The distribution of observations across treatment phases was as follows: induction (22%), consolidation (34%), and maintenance (44%).

Event frequency varied across treatment phases, and was highest during induction (45.1%) and early consolidation (35.7%), and lowest during maintenance (23.4%).

Across all weekly observations: median ANC = 1180/μL (IQR 480–2310); median hemoglobin = 9.4 g/dL (IQR 8.6–10.4); median platelet counts = 184 × 10^9^/L (IQR 95–276); median ANC-CV = 33% (IQR 22–45); and median ANC-TTR = 40% (IQR 20–62).

Event weeks displayed significantly greater marrow suppression and hematologic instability: ANC = 820/μL vs 1460/μL (p < 0.001); ANC-CV = 41% vs 29% (p < 0.001); and ANC-TTR = 26% vs 52% (p < 0.001). Hemoglobin and platelet counts were modestly lower (both p < 0.01), and symptom scores were higher (median = 1 vs 0, p < 0.001). These comparisons are summarized in Table 2.

Symptoms were documented in 51.0% of weekly units; distribution was as follows: score 0 = 49.0%, Score 1 = 35.4%, Score 2 = 15.6%. Weeks with a symptom score of 2 demonstrated a 58.2% event rate compared with 20.1% for score 0.

Model 1 (Hematology-Only Model): Mixed-effects logistic regression identified several independent predictors of 7-day clinical events (Table 3): ANC (per 500/μL decrease): OR 1.17 (95% CI 1.08–1.28), p < 0.001; ANC-CV (per 10% increase): OR 1.09 (95% CI 1.03–1.15), p < 0.001; ANC-TTR (per 10% increase): OR 0.87 (95% CI 0.81–0.93), p < 0.001; hemoglobin (per 1 g/dL decrease): OR 1.10 (95% CI 1.02–1.20), p = 0.014; and platelet counts (per 50 × 10^9^/L decrease): OR 1.06 (95% CI 1.01–1.12), p = 0.026. Model 1 demonstrated good discrimination: AUROC 0.77 (95% CI 0.73–0.80).

**Figure 2:**
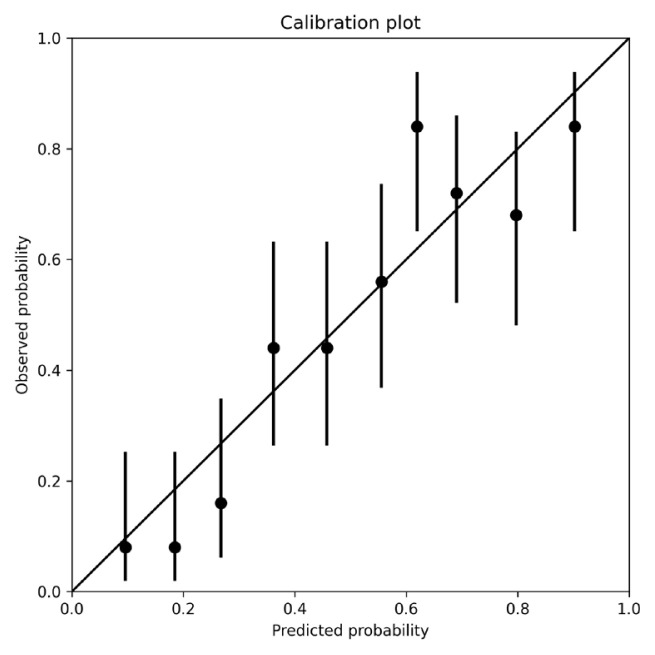
Calibration plot of the final prediction model. Predicted probabilities were grouped into deciles, and the mean observed event rate was plotted against the mean predicted probability. The diagonal line represents ideal calibration.

Model 2 (Hematology + Symptoms): Adding the symptom score improved predictive performance (Table 3): Symptom score (per 1-point increase): OR 1.43 (95% CI 1.30–1.59), p < 0.001. Model 2 achieved an AUROC of 0.83 (95% CI 0.80–0.86). Calibration curves demonstrated close proximity between the predicted and observed probabilities (Hosmer–Lemeshow p = 0.31) (Figure 2). Bootstrap validation demonstrated good calibration, with a calibration slope of 0.92 and an intercept of −0.03, indicating minimal overfitting.

Risk stratification was strong, with event rates ranging from 8.4% in the lowest decile to 58.7% in the highest.

Decision curve analysis (DCA) showed that Model 2 yielded consistently greater net clinical benefit than the Model 1, “treat-all,” and “treat-none” strategies across threshold probabilities of 10–40%, representing clinically relevant decision ranges in pediatric ALL.

Sensitivity analyses conducted using generalized estimating equation (GEE) models yielded comparable population-averaged effect estimates (Table 4), with minimal changes in odds ratios and confidence intervals.

Phase-stratified analyses showed stable AUROCs (0.76–0.84) across induction, consolidation, and maintenance.

## 4. Discussion

This study has examined the predictive value of dynamic hematologic parameters and clinician documented symptoms for forecasting short-term clinical events in children undergoing treatment for ALL. Drawing on data garnered from 1136 weekly observations of 44 patients treated with BFM-based regimens, we demonstrate that temporal hematologic variability, captured through ANC mean, ANC-CV, and ANC-TTR, is strongly associated with adverse clinical events occurring within the subsequent 7 days. The addition of symptom burden further enhanced the model’s predictive performance, yielding a robust and clinically actionable tool.

Our findings build upon a growing body of evidence linking myelosuppression patterns to treatment related morbidity in ALL. There have been numerous studies underscoring the relationship between neutropenia depth and infection risk or treatment interruption [1–4]. However, previous analyses have relied primarily on single time point measurements. In contrast, the current study highlights the importance of week-to-week hematologic stability. Elevated ANC-CV and reduced ANC-TTR were strongly associated with emergency visits, infectious complications, and chemotherapy delays, suggesting that instability, rather than absolute counts alone, may serve as an early physiologic marker of vulnerability. This is consistent with observations that dynamic bone marrow behavior reflects the cumulative effects of pharmacogenomic variability, treatment phase intensity, and adherence patterns [9–12].

Fluctuations in thiopurine and methotrexate metabolism have been documented to contribute to unpredictable myelosuppression [9,15–19]. Genome informed studies have further shown that inter-individual pharmacogenomic heterogeneity significantly impacts marrow toxicity and dose tolerance in ALL [20]. These insights support the biological plausibility of our findings, indicating that hematologic variability likely encapsulates multifactorial determinants that are not captured by fixed laboratory thresholds. Metrics such as ANC-CV and ANC-TTR are derived from routine CBCs and require no specialized testing, making them particularly valuable in settings with no access to universal pharmacogenetic screening tools.

The event frequency in our study (32.3% of weekly observations) was comparable to reports from similar populations, in which febrile neutropenia, infectious episodes, and chemotherapy interruptions remain common during intensive treatment phases [6,21–23]. As expected, event rates were highest during induction and early consolidation, reflecting the intensification of cytotoxic therapy. Although event rates varied across the treatment phases, phase-stratified analyses demonstrated stable model discrimination, suggesting that the predictive value of dynamic hematologic variability is not restricted to intensive treatment phases alone. However, further validation by studies of phase-specific cohorts is warranted.

The incorporation of symptom burden significantly improved model performance (AUROC 0.83). Although the Pediatric PRO-CTCAE provides a validated framework for standardized symptom reporting [16,17], retrospective clinical documentation is often unstructured. Our simplified symptom score, derived directly from clinician notes, proved to be highly informative, demonstrating a strong association between high symptom severity and subsequent clinical events. This reinforces the importance of integrating both objective and subjective indicators into early warning systems for treatment toxicity.

The performance of our combined model compares favorably with earlier prediction tools in pediatric oncology, including models for bacteremia or febrile neutropenia that typically achieve AUROCs in the range of 0.73–0.82 [14,15]. However, direct comparisons of AUROC values from different studies should be approached with caution due to differences in populations, outcomes, and predictor sets.

Several limitations of the present study should be acknowledged, primarily the relatively small number of patients. Although the analysis included 1136 weekly observations, these were clustered within 44 individuals and are therefore not independent. At the observation level, the events-per-variable ratio was acceptable; however, the effective sample size at the patient level was lower. Subsequently, we used mixed-effects modeling, penalized variable selection, and bootstrap internal validation to mitigate overfitting. These strategies are recommended in contemporary prediction model development, particularly when dealing with clustered longitudinal datasets, and external validation in larger and multicenter cohorts is essential to confirm generalizability. Although the number of weekly observations was large, the effective sample size was constrained by the number of patients (clusters). A limited number of predictors were included in the model to reduce overfitting risk and internal bootstrap validation was performed to improve stability; however, external validation with larger multicenter cohorts is necessary.

Second, the retrospective design may introduce documentation variability, particularly in symptom reporting. Such misclassifications likely bias associations toward the null, suggesting that true predictive strength may be even greater. In addition, symptom extraction was performed by a single investigator, and formal inter-rater reliability was not assessed, which may limit reproducibility.

Third, factors such as nutritional status, immune markers, and pharmacogenetic data were not available, but could further enhance predictive accuracy [21–24]. All variables included in the model are routinely recorded in standard pediatric oncology practice, making the approach highly scalable. Finally, although internal validation demonstrated good calibration and discrimination, prospective and external validation will be required before clinical implementation.

In conclusion, dynamic hematologic trajectories combined with clinician-documented symptoms can support the prediction of short-term clinical events in pediatric ALL. The presented model offers promise for risk stratification and proactive supportive care, although future prospective, multicenter validation will be needed ahead of clinical implementation.

## Figures and Tables

**Table 1:** Baseline patient demographic and clinical characteristics.

Variable	Value
Age, years, median (IQR)	6.1 (3.3–10.4)
Male sex, n (%)	23 (52.3)
Immunophenotype	
B-cell ALL, n (%)	37 (84.1)
T-cell ALL, n (%)	7 (15.9)
BFM risk group	
Standard-risk, n (%)	20 (45.5)
Intermediate-risk, n (%)	17 (38.6)
High-risk, n (%)	7 (15.9)
WBC at diagnosis, ×10^9^/L, median (IQR)	11.8 (4.7–45.2)
End-induction MRD ≥10^−4^, n (%)	9 (20.5)
Median follow-up, months, median (IQR)	14.1 (10.0–19.6)
Total weekly observation units, n	1136
Relapse within 2 years, n (%)	4 (9.1)
All-cause mortality, n (%)	2 (4.5)

**Table 2:** Hematologic parameters and symptom burden in event vs nonevent weeks.

Parameter	Event weeks (n = 367)	Nonevent weeks (n = 769)	p-value
ANC (/μL), median (IQR)	820 (330–1560)	1460 (650–2580)	<0.001
ANC-CV (%), median (IQR)	41 (29–54)	29 (20–40)	<0.001
ANC-TTR (%), median (IQR)	26 (10–45)	52 (30–71)	<0.001
Hemoglobin (g/dL), mean ± SD	9.1 ± 1.2	9.6 ± 1.1	0.006
Platelets (×10^9^/L), median (IQR)	168 (92–242)	197 (110–306)	0.008
Symptom score, median (IQR)	1 (0–2)	0 (0–1)	<0.001

**Table 3:** Mixed-effects logistic regression models predicting 7-day clinical events.

Predictor	Model 1 (hematology only) OR (95% CI)	p-value	Model 2 (hematology + symptoms) OR (95% CI)	p-value
ANC (per 500/μL decrease)	1.17 (1.08–1.28)	<0.001	1.14 (1.06–1.24)	<0.001
ANC-CV (per 10% increase)	1.09 (1.03–1.15)	<0.001	1.07 (1.02–1.13)	0.003
ANC-TTR (per 10% increase)	0.87 (0.81–0.93)	<0.001	0.89 (0.83–0.95)	<0.001
Hemoglobin (per 1 g/dL decrease)	1.10 (1.02–1.20)	0.014	1.08 (1.01–1.17)	0.022
Platelet counts (per 50×10^9^/L decrease)	1.06 (1.01–1.12)	0.026	1.05 (1.00–1.11)	0.041
Symptom score (per 1-point increase)	–	–	1.43 (1.30–1.59)	<0.001
AUROC	0.77	–	0.83	–

**Table 4:** Sensitivity analysis using generalized estimating equations (GEE). Population-averaged odds ratios (ORs) for 7-day clinical events.

Predictor	GEE OR (95% CI)	p-value
ANC (per 500/μL decrease)	1.15 (1.06–1.25)	<0.001
ANC-CV (per 10% increase)	1.08 (1.02–1.14)	0.005
ANC-TTR (per 10% increase)	0.88 (0.82–0.94)	<0.001
Hemoglobin (per 1 g/dL decrease)	1.07 (1.00–1.16)	0.048
Platelet counts (per 50×10^9^/L decrease)	1.05 (1.00–1.10)	0.041
Symptom score (per 1-point increase)	1.39 (1.27–1.53)	<0.001

## Data Availability

Data supporting the findings of this study are available from the corresponding author upon reasonable request
